# Micro-Raman spectroscopic analysis on natural carbonates: linear relations found via biaxial plotting of peak frequencies for cation substituted species

**DOI:** 10.1007/s44211-022-00119-1

**Published:** 2022-05-18

**Authors:** Shu-hei Urashima, Tomoya Nishioka, Hiroharu Yui

**Affiliations:** 1 Department of Chemistry, Faculty of Science, Tokyo University of Science, 1-3 Kagurazaka, Shinjuku, Tokyo 162–8601 Japan; 2 Water Frontier Research Center, Research Institute for Science & Technology, Tokyo University of Science, 1-3 Kagurazaka, Shinjuku, Tokyo 162–8601 Japan

**Keywords:** Carbonate, Solid solution, Raman, Non-destructive, Biaxial plotting

## Abstract

Carbonates are ubiquitous minerals carrying important information on aqueous environments where they precipitated on the Earth and space. While their ideal chemical formulae are denoted as simple as MCO_3_ or M_1_M_2_(CO_3_)_2_ (M: metal cations), natural carbonates generally form solid-solution series and their compositions deviate from the ideal formulae. Since their cation composition due to the substitution provides a sensitive indicator for chemical and thermodynamic environments of aqueous solutions where they precipitated, their composition analysis has been widely carried out from the environmental/geochemical/astrochemical aspects. However, in widely used back-scattered electron and energy dispersion X-Ray analyses, samples should be generally sliced and/or their surface be polished prior to the measurements. For analyzing rare samples with small sizes, such as ones sampled from deep-sea and/or meteorites and asteroids, a non-destructive method without any pretreatments has been strongly desired. Here, a novel analytical method for discriminating various carbonates with Raman micro-spectroscopy is demonstrated, showing that the biaxial plot of the peak frequencies of their lattice modes linearly moves upon partial substitution of the cations. The cation substitution leads to linear movement in the biaxial map, and the slopes of the movement were different for Mg^2+^-Fe^2+^ and Mn^2+^-Fe^2+^ substitutions. This finding suggests that the micro-Raman analysis would be a non-destructive analytical method for evaluating the relative amount of Mg^2+^, Fe^2+^, and Mn^2+^ in dolomite-ankerite-kutnohorite solid-solution series, as well as Mg^2+^/Fe^2+^ ratio for magnesite-breunnerite-siderite. It would be helpful for analyzing the present and past terrestrial and cosmochemical environments.

**Graphical abstract:**

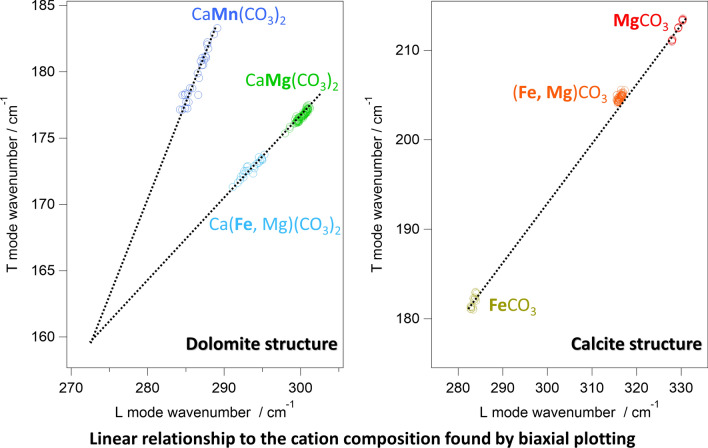

**Supplementary Information:**

The online version contains supplementary material available at 10.1007/s44211-022-00119-1.

## Introduction

Carbonate minerals are ubiquitous in the present and past aqueous-related environments in the Earth, meteorites, and their parent bodies as sedimentary rocks [1–3]. In the Earth, it is believed that the carbonates in the sedimentary rock noticeably increase soil strength [4]. In addition, they are also known as the biggest natural storage of CO_2_, which is deeply related to the carbon cycle in the environments [5–7]. Thus, the use of carbonates as carbon storage has attracted much attention also from the viewpoint of artificial carbon fixation to prevent global warming [8]. Furthermore, since carbonates record the past aqueous environment when and where they were formed, natural carbonates including biogenetic ones have been extensively studied in geochemical and cosmochemical sciences [9, 10]. For example, their existence on meteorites gives direct evidence for the past aqueous environments on their parent bodies because fluidic water is inevitable to precipitate them. Therefore, analyses of the carbonates have been very important in various aspects of environmental chemistry, geoscience, and astrochemistry.

However, even the determination of their chemical composition of the natural carbonates is not easy. One of the reasons is that, especially in dolomite group carbonates, the cations are partially substituted with others while their ideal chemical formulae are simple. For example, Mg^2+^ in natural dolomite CaMg(CO_3_)_2_ is known to be substituted with Fe^2+^ and Mn^2+^. The Fe^2+^-rich dolomite is called ankerite, and the Mn^2+^-rich dolomite is called kutnohorite. The end member chemical formulae of ankerite and kutnohorite are thus CaFe(CO_3_)_2_ and CaMn(CO_3_)_2_, respectively. They form a solid-solution series with continuous substitution of Mg^2+^–Fe^2+^–Mn^2+^ [11, 12]. In addition, ideal CaFe(CO_3_)_2_ is especially unstable so that it is obtainable only by artificial synthesis [13]. Fe^2+^ composition of natural ankerite is about 70% at most [11], and hence it is sometimes regarded as ferroan dolomite [14, 15]. Similarly, magnesite (MgCO_3_)—breunnerite [(Mg, Fe)CO_3_]—siderite (FeCO_3_) forms a solid solution series by continuous substitution of Mg^2+^ and Fe^2+^ [16] (Note that “breunnerite” is not an accepted name by International Mineralogical Association and it should be called as ferroan magnesite. However, the term “breunnerite” is familiar especially to astrochemists and widely used even in very recent works [17–19]. In this paper, “breunnerite” is used for this reason). Such a continuous and wide range of substitution is one of the characteristic feature of dolomite group carbonates. In natural calcite and magnesite, on the other hand, it is well known that Ca–Mg substitution very limitedly occurs (*e.g.* about 5% of Ca is substituted with Mg). The difference can be found also from the viewpoint of substitution structure. While two cations in dolomite are in well-defined positions, Ca in calcite are only randomly substituted with Mg. Such a different manner of substitution leads to different space groups for dolomites (R-3) and calcites (R-3c). The first reason for the difficulty of chemical composition determination is that, in summary, the distinguishment of carbonates as *e.g.* dolomite, ankerite, or kutnohorite is not enough for dolomites because of the wide-range and continuous cation substitution. The second reason is that natural carbonates often exist as small crystals with micrometer-scale and are inhomogeneously scattered on matrix rocks. Spatial resolution with micrometer-scale is necessary. These are why cation composition analysis of carbonates, especially in a non-destructive manner, has remained a challenging issue.

Because of imaging applicability with high spatial resolution, back-scattered electron (BSE) and/or energy-dispersive X-ray (EDX) techniques have been widely employed for the composition analysis of various kinds of carbonates [1, 3, 20]. They have strong merits in spatial resolution for the analysis of small carbonates crystals inhomogeneously distributed on the matrix rocks, but they often require the samples to be sliced and/or their surface to be polished. Such pretreatments sometimes induce unfavorable mechanical/thermal damage to the samples. In addition, they are sometimes even difficult for extremely rare samples such as micrometer-scale cosmochemical samples, *e.g.*, highly aqueously-altered carbonaceous chondrites, samples directly captured from planetary bodies such as the moon and asteroids, and ones captured from the deep-sea. The pretreatments and/or any other destructive analytical techniques should be the last resort for them.

From the viewpoint of a non-invasive and non-destructive manner, X-ray fluorescence (XRF) is useful for the elemental analysis of metal cations in carbonates. It surely gives information about the elemental composition with moderate spatial resolution, but it is sometimes difficult to distinguish the signal from the elements in carbonates and in co-existing other minerals of matrix rocks. For example, Mg^2+^ are ubiquitous in the matrix rocks as serpentine (Mg_3_Si_2_O_5_(OH)_4_), so that Mg^2+^ composition in magnesite (MgCO_3_) is difficult to be evaluated only by XRF. Further, the fluorescent peak intensity of XRF often suffers from matrix effects by co-existing elements. Therefore, the establishment of a nondestructive, pretreatment-free, and generally applicable method for carbonate analysis with micrometer-scale spatial resolution has been strongly desired.

To overcome the problems in the conventional methods for discriminating and analyzing various kinds of carbonates, the applicability of Raman micro-spectroscopy was focused on in the present study. Raman spectroscopy is essentially non-destructive, and it can be readily applicable to imaging analysis with micrometer resolution by combining with a microscope. Further, the working distance of the objective lenses reaches centimeter-scale, allowing us to directly measure the inhomogeneous samples having surface roughness without any surface polishing pretreatments. Despite these advantages, spectral databases of Raman spectra for carbonates are still limited especially for their solid-solution systems [2, 15, 21–30]. For example, Raman spectra of natural breunnerite have never been reported and they could not be found even in RRUFF spectral database [31]. As far as we know, Raman spectra of artificially synthesized ferromagnesites [(Mg,Fe)CO_3_] are the only available data which might be regarded as breunnerite [30].

According to the previous reports on Raman spectroscopy on carbonates, there are four strong bands and two weak bands appearing in the Raman spectra. Two of the strong bands are lattice modes having E_g_ symmetry with translational (T) and liberational (L) motion of CO_3_^2−^ ions. Hereafter they are simply called T and L modes, respectively. The other strong bands are bending and stretching vibration of CO_3_^2−^. They possess E_g_ and A_1g_ symmetry, respectively, and hereafter they are called ν_4_ and ν_1_ modes according to nomenclature in the literature [22, 24, 26, 27, 30]. Since these Raman-active modes originate from the vibrational motion of CO_3_^2−^ moieties, the Raman spectra of the carbonates show similar spectral patterns regardless of the kind of carbonates. The vibrational frequencies of T, L, ν_4_, and ν_1_ bands are about 200, 300, 700, and 1100 cm^−1^, respectively. In the case of single cation carbonates, the substitution of the cation from Ca^2+^ to Fe^2+^, Mg^2+^, or Mn^2+^ leads to about 30–60 cm^−1^ of blue shift for T and L modes [2, 21–23]. Although these peak shifts are large enough to be distinguished in the spectra, it still remains difficult to determine the various cation composition of the solid-solution series of the carbonates. Kim et al*.* and Rividi et al*.* independently tried to evaluate the cation composition for the solid solutions from Raman and infrared spectra [15, 24]. While they found a trend between the relative amount of Mg^2+^ and peak frequencies of T and L modes, the unique determination of Mg–Fe^2+^–Mn^2+^ composition by Raman micro-spectroscopy has not been achieved.

In the present study, Raman spectra of a variety of single- and multi-cation carbonates, including natural breunnerite, were measured to find a trend in various carbonates including Ca^2+^, Mg^2+^, Fe^2+^, and Mn^2+^. They are the representative divalent metal cations for forming various carbonates found in both terrestrial and cosmochemical environments. In addition to simply distinguishing the kind of carbonates, a biaxial plotting of the vibrational frequencies was proposed to uniquely discriminate their solid-solution series with varying Mg^2+^-Fe^2+^-Mn^2+^ composition from the Raman spectra.

## Experiment

**Fig. 1:**
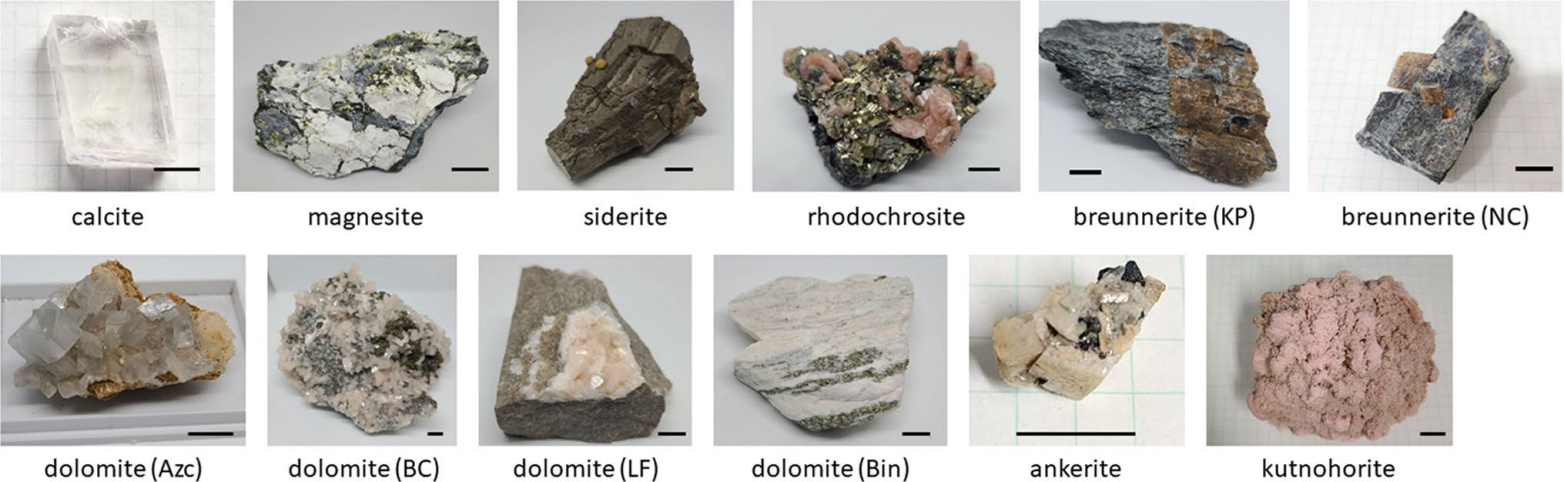
The samples used in this study. The scale bars correspond to 1 cm

**Fig. 2:**
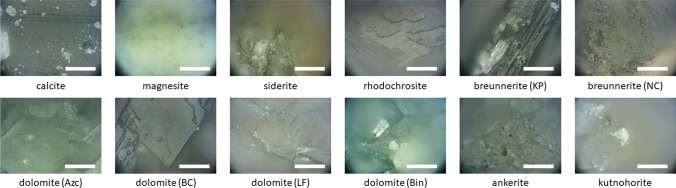
Microscopic pictures of the samples. The scale bars correspond to 50 μm

The carbonates prepared were calcite, magnesite, siderite, rhodochrosite, dolomite, breunnerite, ankerite, and kutnohorite. The mines for each mineral are summarized in Table 1. Some of the minerals were collected from a couple of different locations. Such the samples are hereafter called with the locations as *e.g.* dolomite (Azc) and dolomite (BC) as described in Table 1. To obtain fresh surfaces, millimeter-sized pieces were collected from each sample with a chisel and a hammer prior to the measurement. Macroscopic and microscopic pictures of the minerals are shown in Figs. 1 and 2, respectively.

**Table 1:** The carbonates prepared in this study

Mineral	Location
Calcite	A mine in Mexico. Further information was unavailable
Magnesite	Goat Hill Magnesia Quarries, West Nottingham Township, State Line Chromite Mining District, Pennsylvania, USA
Siderite	El Potosi Mine, Sta. Eulalia, Chihuahua, Mexico
Rhodochrosite	Eagle Mine, Colorado, USA
Breunnerite (KP)	Krasnaya Polyana, Chebarkulskiy District of Urals, Russia
Breunnerite (NC)	Novo-Cheremshanskoye field, Russia
Dolomite (Azc)	Azcarate Quarry, Eugui, Esteribar, Navarre, Spain
Dolomite (BC)	Butler Country, Missouri
Dolomite (LF)	La Farge Quarries, NewYork, USA
Dolomite (Bin)	Binntal, Switzerland
Ankerite	Eagle Mine, Colorado, USA
Kutnohorite	Wissels mine, Hotazel, Kalahari manganese field, Northern Cape, South Africa

To check the homogeneity of the samples, the Raman spectra were measured at least 10 different spots. In the data set, unassigned minerals other than carbonates were found only at a few spots, and they were excluded from the analysis.

The Raman measurements were performed with Raman-11i (Nanophoton), which is combined with Eclipse Ti (Nikon) microscope. An objective lens of Nikon Plan Fluor (40x, NA 0.75, WD 0.66) was used for focusing the incident laser on the sample and backscattered light was collected by the same objective lens. The excitation wavelength was 532 nm, and the power was 10 mW at the sample face. The spectra were measured in 120-s exposure and averaged 2 times. The slit width for the entrance of the spectrometer was 50 μm, and a grating of 1200 grooves/mm was used. With this measurement condition, the spectra were recorded from about -30 to + 1270 cm^−1^ at every 1.1 cm^−1^. Bandwidth for the standard Si peak (at 520.6 cm^−1^) was observed as 4–5 cm^−1^ in full-width-of-half-maximum (FWHM), which indicates the spectral resolution was at most 4–5 cm^−1^.

To precisely determine the peak position regardless of the pixel spacing of 1.1 cm^−1^, each peak was fitted by the Lorentzian function after baseline correction. While the spectral resolution was estimated as ~ 5 cm^−1^ at most, the fitting analysis enables us to determine the peak frequency with higher accuracy than the resolution. According to literature, even if each peak was broadened by instrumental function, the peak frequency of a single band can be determined with an accuracy of 1/10 time of the pixel spacing by the fitting when the signal-to-noise (S/N) ratio is better than 10 [32]. As shown in the Results section, the S/N ratio of our data was typically higher than 10. Therefore, since the pixel spacing was 1.1 cm^−1^, the peak frequency can be determined as precise as ~ 0.1 cm^−1^. For the Lorentzian fitting, only the region of ± 5 pixels from the apparent peak top was fitted. This is because some of the peaks had an asymmetric tail. The region of ± 5 pixels (~ 10 cm^−1^) roughly corresponded to the FWHM of the carbonate bands so that the effect of the tail was practically negligible in this region. The baseline correction was performed because some of the spectra have fluorescent backgrounds, which should alter apparent peak frequency (peak dragging). For determining the peak position of each mode as accurately as possible, the baseline was individually calculated for each band. The baseline was defined as a linear function which connects the spectral points at ± 50 cm^−1^ from the apparent peak frequency. While the background was not linear in the whole spectral region, the linear function was of good approximation for the region of only 100 cm^−1^ around each peak. Note that the tail of the bands was negligible at ± 50 cm^−1^ far from the peak.

For T and L modes, the spectral frequency was calibrated with Raman bands of a sulfur flake (153.8, 219.1, and 473.2 cm^−1^) [33] and of a silicon wafer (520.6 cm^−1^) [34]. This calibration was performed by assuming the pixel and the light wavelength are related to a linear function. For ν_4_ and ν_1_ modes, the peak frequency was calibrated only by that of the silicon wafer. This calibration is simply performed by taking frequency-offset as the silicon band to be at 520.6 cm^−1^. This is because all the calibration bands of sulfur and silicon are in the low-frequency region (< 520 cm^−1^) so that the linear fit should be no longer accurate at a higher frequency. Despite the disadvantage for accuracy in the high-frequency region, the calibration with multiple bands of sulfur and silicon provides very high accuracy for vibrational frequency of T and L modes. As shown in Figure S1 in the Supporting Information (SI), the standard deviation for repeating measurements was less than 0.25 cm^−1^ in this region.

## Results

**Fig. 3:**
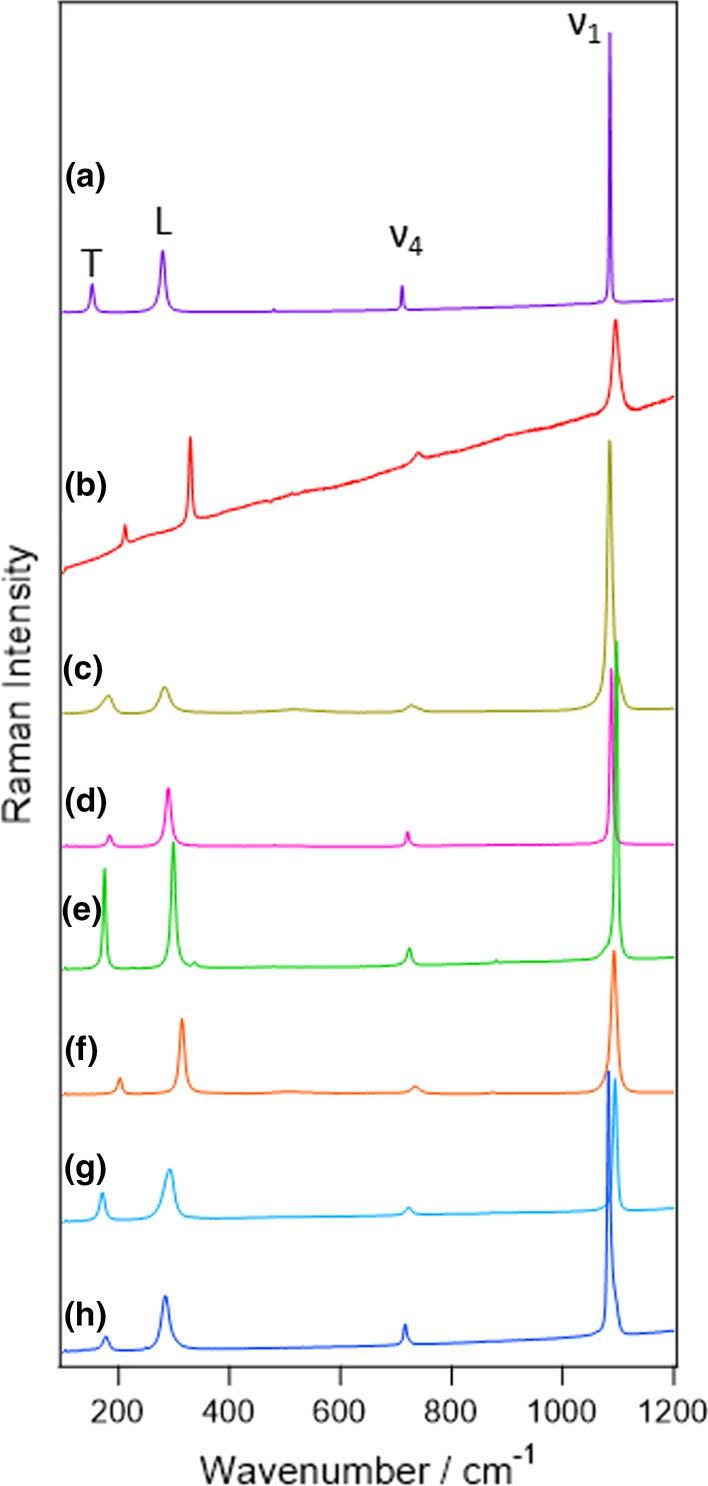
Typical Raman spectra of the carbonates: **a** calcite, **b** magnesite, **c** siderite, **d** rhodochrosite, **e** dolomite (BC), **f** breunnerite (KP), **g** ankerite, **h** kutnohorite. The Raman shift was calibrated by that of the silicon wafer. Those for dolomite and breunnerite are shown only for those from 1 mine because no difference was found

Typical spectra obtained for each sample are shown in Fig. 3. As described in the Introduction, four distinct bands at around 200 (T), 300 (L), 700 (ν_4_), and 1100 cm^−1^ (ν_1_) were observed for all samples. This similarity indicates that the samples prepared were indeed carbonate minerals. The peak frequencies for each mineral are also summarized in Table 2. For single cation carbonates, magnesite had the highest vibrational frequency. Siderites, rhodochrosite, and then calcite followed it. This order is consistent with previous reports [2, 15, 21–23] while the peak frequencies slightly deviated from them. The modest difference might be owing to different calibration schemes and/or slight variations of the cation composition. Among four normal modes, the frequencies of T and L modes were more sensitively dependent on the mineral than the others. This is because T and L modes are the lattice modes while the others are local vibrational modes in the carbonate ion.

**Table 2:** Peak frequencies (mean ± standard deviation) of the carbonates studied

	End member formulae	T	L	ν_4_	ν_1_
Calcite	CaCO_3_	154.6 ± 0.9	280.9 ± 0.9	711.6 ± 0.8	1086.0 ± 0.8
Magnesite	MgCO_3_	212.5 ± 1.0	329.3 ± 1.0	738.2 ± 1.1	1094.4 ± 1.0
Siderite	FeCO_3_	181.9 ± 0.7	283.4 ± 0.5	728.9 ± 3.7	1084.7 ± 0.2
Rhodochrosite	MnCO_3_	184.8 ± 0.4	289.9 ± 0.5	721.2 ± 0.5	1087.7 ± 0.2
Breunnerite (KP)	(Mg, Fe)CO_3_	204.8 ± 0.4	316.2 ± 0.4	734.8 ± 0.3	1092.2 ± 0.3
Breunnerite (NC)	(Mg, Fe)CO_3_	204.6 ± 0.4	316.3 ± 0.5	735.2 ± 0.4	1092.7 ± 0.5
Dolomite (Azc)	CaMg(CO_3_)_2_	176.7 ± 0.5	300.2 ± 0.8	725.2 ± 0.4	1098.5 ± 0.5
Dolomite (BC)	CaMg(CO_3_)_2_	176.7 ± 0.4	300.2 ± 0.4	724.5 ± 0.7	1097.8 ± 0.9
Dolomite (LF)	CaMg(CO_3_)_2_	176.4 ± 0.2	299.6 ± 0.3	724.3 ± 0.7	1097.4 ± 0.7
Dolomite (Bin)	CaMg(CO_3_)_2_	176.9 ± 0.4	300.6 ± 0.7	725.6 ± 0.2	1099.1 ± 0.2
Ankerite	CaFe(CO_3_)_2_	172.7 ± 0.7	293.5 ± 1.2	721.5 ± 1.3	1093.6 ± 1.3
Kutnohorite	CaMn(CO_3_)_2_	179.7 ± 1.9	286.4 ± 1.4	717.2 ± 1.1	1083.5 ± 1.3

**Fig. 4:**
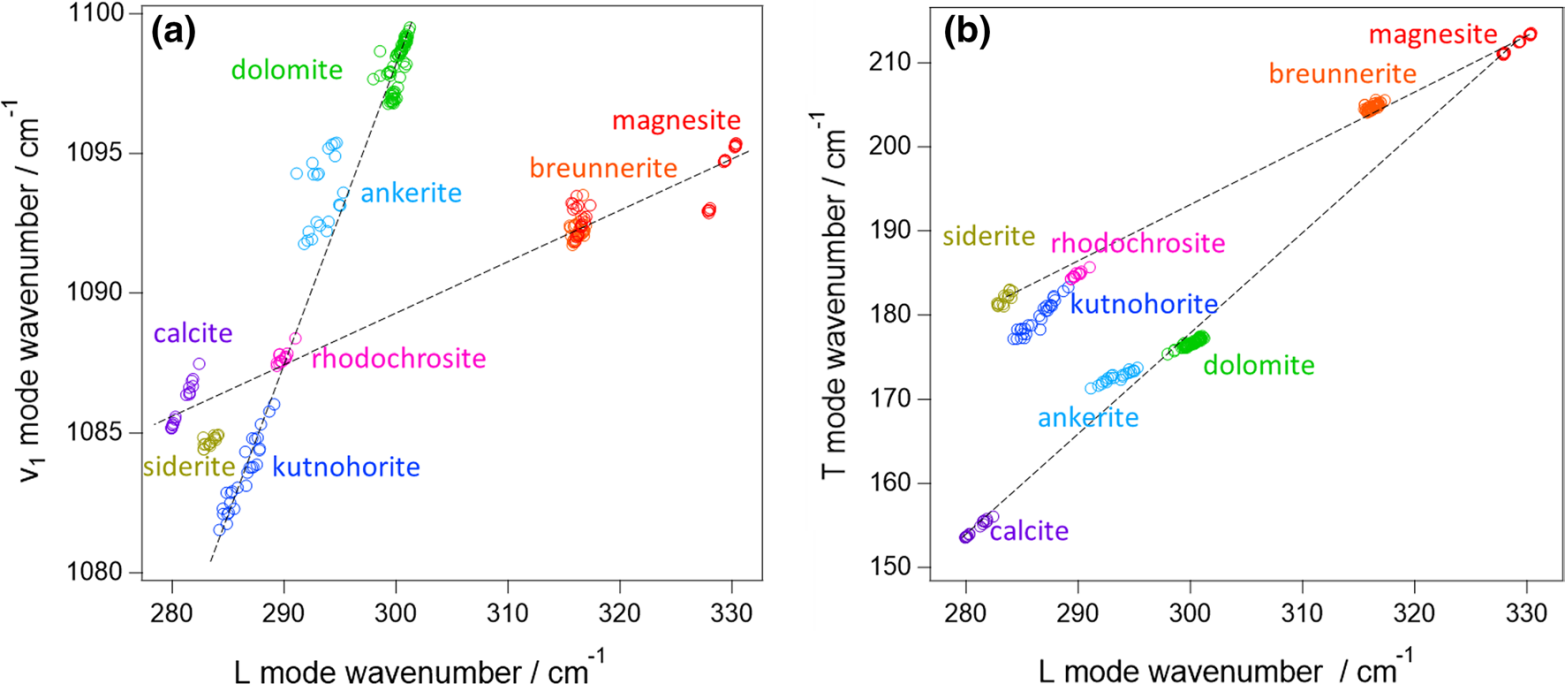
Biaxial plot of peak frequencies for **a** ν_1_ and L modes, and **b** T and L modes. The broken lines are eye guides

To visualize the cation-dependent frequency shift, the vibrational frequencies were biaxially plotted (Fig. 4). Importantly, each mineral was well separated in both of ν_1_–L and T–L biaxial plots (Figs. 4a and b, respectively), enabling the cation identification from the Raman spectra. Note that, while 4 dolomites and 2 breunnerites were prepared in this study, their peak frequencies were not dependent on the mine (Fig. S2 in the SI). In ν_1_–L biaxial plot (Fig. 4a), the points for calcite structure (single salt carbonates and breunnerite) were on a single line as well as those for dolomite structure (dolomite, ankerite, and kutnohorite) located on another line. For dolomite series, cation substitution gave rise to high-frequency shift in the order of Mn^2+^  < Fe^2+^  < Mg^2+^. The order for the calcite series was Ca^2+^  ~ Fe^2+^  < Mn^2+^  < Mg^2+^, in which the order of Fe^2+^ and Mn^2+^ are inverted from the case of the dolomite series. On the other hand, neither calcite nor dolomite series made a single line in T–L biaxial plot (Fig. 4b). Instead, the points for double salts and solid-solutions located in-between those for two single salts: *i.e.*, dolomite [CaMg(CO_3_)_2_] was located between calcite (CaCO_3_) and magnesite (MgCO_3_), and breunnerite [(Mg,Fe)CO_3_] was in-between siderite (FeCO_3_) and magnesite (MgCO_3_).

## Discussion

For single cation carbonates, the vibrational frequencies of individual modes had been related to their ionic radii and nearest neighbor distance [2, 15, 21–23]. While a nearly linear relation was found between them, its correlation coefficient was ~ 0.9 and ~ 0.7 for T and L modes, respectively. These low correlation coefficients hinder us to identify the carbonates including multi-cation species. Because multi-cation carbonates are often found on meteorites and other geoscientific samples, the unambiguous assignment using the biaxial plot (Fig. 4) is highly advantageous. However, because the cation ratio of solid-solution series is continuously variable in nature, it is desired to determine the chemical composition more precisely, not to simply distinguish the kind of carbonates. For this purpose, the “movement” of the points in the biaxial map upon the cation substitution is discussed.

As shown in Fig. 4, each carbonate is clearly separated in both of T–L and ν_1_–L maps. However, in ν_1_–L map (Fig. 4a), each calcite group and dolomite group forms a single line. This means that Mn^2+^–Fe^2+^ substitution and Fe^2+^–Mg^2+^ substitution lead to the movement with the same slope on the biaxial plot, and hence it is difficult to uniquely estimate the amount of each cation from ν_1_–L plot. In T–L plot (Fig. 4b), on the other hand, dolomite, ankerite, and kutnohorite formed a triangle, not a line. The use of T–L plot is thus more promising for uniquely estimating cation composition. Furthermore, the frequency shift of ν_1_ mode should correspond to the change in the local environment around the CO_3_^2−^, not the global change. While global cation substitution must also change the local environment (that is why each carbonate was well separated in Fig. 4a), the frequency shift of ν_1_ mode does not always mean a change in the global structure. In fact, calcite in Fig. 4a is widely spread while a significant amount of cation substitution is not expected for calcite. Because the spread is mainly in ν_1_ mode frequency, the wide distribution in Fig. 4a might be due to local defects presumably around the randomly substituted Mg. While the origin of the spread is unclear, it can be concluded that it is difficult to directly relate the cation composition with ν_1_ mode frequency. Therefore, T–L plot will be mainly discussed.

As described in the Results section, the plots of dolomite were located on a line which connects those of calcite and magnesite. This implies that the substitution of the cation leads to linear movement in the T–L map. In fact, the same relation can be found for the series of siderite, breunnerite, and magnesite. More importantly, each carbonate linearly spread in the T–L map. The distribution range in each mineral was ~ 5 cm^−1^ for example for L mode, and it was broader than the experimental deviation (± 0.25 cm^−1^). The linear distribution is thus attributable to the heterogeneity of cation composition in each sample. Because almost all dolomites showed a large spread in the biaxial plot, the heterogeneity in a single chip of each crystal is considered a general feature of carbonates when we probe it with micrometer-scale spatial resolution.

To analyze the cation substitution in detail, the slopes of the lines connecting the single-cation carbonates are considered at first. By using the mean of each vibrational frequency, they were calculated as 1.20, 0.67, 10.92, 3.36 for calcite-magnesite, siderite-magnesite, calcite-siderite, and calcite-rhodochrosite lines, respectively. If partial substitution of the cation leads to linear movement in the biaxial map, the same slope should be found in solid-solution series. However, the slope of the linear spread in dolomite was 0.60. This was noticeably different from that of calcite-magnesite line (1.20) although dolomite locates between calcite and magnesite. This is understandable because dolomite is a double salt, not a solid solution of Ca^2+^ and Mg^2+^. As described in “Introduction”, the drastic substitution of Ca^2+^ and Mg^2+^ (calcite to magnesite) is not expected in nature. Instead, dolomite forms solid-solution series with ankerite by continuous substitution of Mg^2+^ to Fe^2+^. In fact, the points for ankerite also linearly spread in the biaxial map with a slope of 0.52. Because the points for dolomites and ankerite were essentially on the same line, linear distribution with the slope of ~ 0.5 is attributable to Mg^2+^/Fe^2+^ substitution. This slope was similar to that of siderite-breunnerite-magnesite series (~ 0.7) because both were related to Mg^2+^/Fe^2+^ substitution. The slight difference is presumably because one half of the cation is always occupied with Ca^2+^ in dolomite-ankerite solid solution series while the cation is fully substituted from Fe^2+^ with Mg^2+^ in siderite-breunnerite-magnesite series. It is noteworthy that the distribution line of dolomites points crosses the calcite-magnesite line. Because the frequency shifts to the higher side upon the substitution from Fe^2+^ to Mg^2+^, the cross of the line indicates that the end member dolomite (CaMg(CO_3_)_2_) is not perfectly on the calcite-magnesite line. While end member dolomite was expected to locate on the calcite-magnesite line as a first approximation, this approximation was too simplified for the structural change from calcite (single salt) to dolomite (double salt). The linear relation is thus adaptable only for solid-solution series in a strict sense. Note that dolomite forms solid-solution series also with kutnohorite, whose end-member formula is CaMn(CO_3_)_2_. In reality, dolomite-ankerite-kutnohorite solid-solution series cannot be separated from each other. The existence of Mn^2+^ in dolomite and ankerite should not be neglected in the analysis. However, spectral evidence with Mn^2+^ substitution could not be found for dolomite and ankerite. This might be because Mn^2+^ was minor in dolomites and ankerite prepared in this study. Spectral change which corresponds to a slight increase/decrease of Mn^2+^ should be overwhelmed by that corresponding to Mg^2+^/Fe^2+^ substitution.

As for kutnohorite, it is located in-between calcite and rhodochrosite in the biaxial plot. As in the case of dolomite, kutnohorite plots were not perfectly on the calcite-rhodochrosite line. The slope of the kutnohorite distribution was 1.3. Because this slope is far from that for Fe^2+^/Mg^2+^ substitution line (~ 0.5), the linear distribution in kutnohorite is assignable to variation of Mn^2+^ / (Fe^2+^, Mg^2+^) ratio. Further, because the plot should get close to dolomite if Mn^2+^ is substituted with Mg^2+^, the linear distribution found in kutnohorite is assignable to Mn^2+^/Fe^2+^ substitution. Here, it should be again stressed that the linear distribution in kutnohorite was assignable to the inhomogeneity of Mn^2+^ composition in the rock. The distribution of the peak frequency was especially wide for kutnohorite in comparison with those for other carbonates (Table 2). While it is ambiguous why kutnohorite is more heterogeneous than other carbonates, the wide spreading of kutnohorite helps us confidently assign the line with the slope of ~ 1.3 to Mn^2+^/Fe^2+^ substitution. The assignment would be further proved if more Fe-rich kutnohorite could be prepared as Mg^2+^/Fe^2+^ substitution line was constructed by using dolomite and ankerite (ferroan dolomite).

**Fig. 5:**
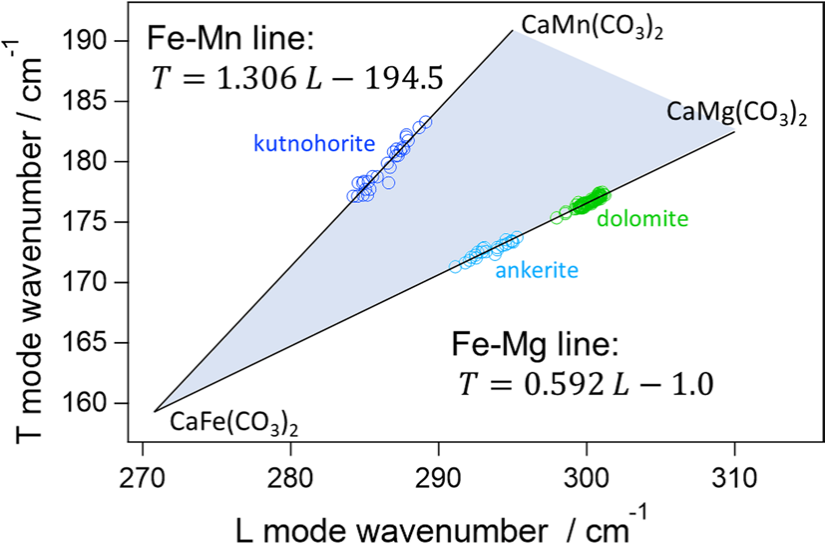
Ternary Raman diagram for Mg–Fe^2+^–Mn^2+^ composition in dolomite solid-solution series. The coefficients for Fe–Mg line were obtained by simultaneous linear fitting for ankerite and dolomite

As a summary of the dolomite group analysis, we propose a Mg^2+^-Mn^2+^-Fe^2+^ ternary Raman diagram for the dolomite group as shown in Fig. 5. Because solid solution leads to the linear, continuous movement for the biaxial plot, any composition of dolomite-ankerite-kutnohorite series should mark the plot inside the triangle. Note that an assumption was necessary for the position of CaFe(CO_3_)_2_ because end member carbonates were not prepared in this study. Especially CaFe(CO_3_)_2_ is unstable and it had never been found in nature. Therefore, it is assumed that the end member CaFe(CO_3_)_2_ locates at the intersection of the ankerite-dolomite line and kutnohorite line: (L mode, T mode) = (270.8 cm^−1^, 159.3 cm^−1^). These assumptions might not be very accurate so that the end-member positions should be replaced with the real values in the future. This is possible by using artificially synthesized carbonates. Unfortunately, the elemental component of the samples prepared in the present study is currently unknown, hindering us to quantify this ternary map. However, there are a few studies in which Raman frequency and elemental components are reported for the dolomite-ankerite series [15, 24]. As shown in Table S1 and Figure S3 in the SI, a trend that Mg-rich carbonates showed higher frequency was found also in the literature data. By comparing the literature and the data obtained in the present study, Mg / (Mg + Fe) ratio of ankerite prepared here is expected to be about 0.7. Note that spectral resolution and calibration procedure in the literature were different from those in the present study so that the absolute value of Mg / (Mg + Fe) ratio would have some uncertainty. Comparison with elemental analysis (such as X-ray fluorescence, BSE, or energy-dispersive X-ray measurements) and Raman spectra should be performed for better quantification in future work. It is especially important for kutnohorite, which is rarely found in the literature. Despite these limitations at the present stage, the linear relations found in Figs. 4 and 5 can be a good compass for the intact analysis of the carbonates on meteorites.

## Conclusions

Raman spectra of terrestrial carbonates mined at 12 different locations are measured. The carbonate type can be unambiguously identified by biaxially plotting the vibrational frequencies of the lattice modes. Importantly, in addition to simply distinguishing the type of carbonate, the linear movement in the biaxial map was found upon cation substitution. For dolomite-ankerite-kutnohorite solid solution series, Mg^2+^-Fe^2+^ and Fe^2+^-Mn^2+^ substitutions gave rise to different slopes of linear movement in the biaxial map of the lattice modes. This finding suggested that Mg^2+^-Fe^2+^-Mn^2+^ ratio of this solid solution series can be uniquely estimated from their Raman spectra. A similar linear relationship was found also for Mg^2+^/Fe^2+^ substitution in siderite-breunnerite-magnesite solid solution series. While further quantitative analysis should be carried over to future work, the present study would open the way to analyze carbonates in a non-destructive manner without any pretreatments. The combination with microscopes is helpful also for inhomogeneous and small crystals of carbonates, which are often found in cosmochemical samples and terrestrial rocks. Therefore, the present study would help to retrieve important information on the changes in aqueous environments that lead to the origins of the Earth and life, as well as information on the carbon circulation and the climate changes in the long-time history of the Earth.

## Supplementary Information

Below is the link to the electronic supplementary material.

Supplementary file1 (DOCX 132 KB)

## Data Availability

The datasets generated and/or analyzed during the current study are available from the corresponding author on reasonable request.
