# Supplementary material for: Micro-Raman spectroscopic analysis on natural carbonates: linear relations found via biaxial plotting of peak frequencies for cation substituted species
Source: Anal Sci. 2022 May 18;38(7):921–9. doi: 10.1007/s44211-022-00119-1 (PMC9206923; doi:10.1007/s44211-022-00119-1)
Supplement: Supplementary file 1 — Supplementary file1 (DOCX 132 KB) [file 44211_2022_119_MOESM1_ESM.docx]

*Supporting Information*

*for*

**Micro-Raman spectroscopic study of Natural Carbonates: Linear relation on biaxial peak frequency plot for cation substituted species**

Shu-hei Urashima^a,b^, Tomoya Nishioka^a^, Hiroharu Yui^a,b^*

^a^ *Department of Chemistry, Faculty of Science, Tokyo University of Science,1-3 Kagurazaka, Shinjuku, Tokyo 162–8601, Japan.*

^b^ *Water Frontier Research Center, Research Institute for Science & Technology, Tokyo University of Science, 1-3 Kagurazaka, Shinjuku,Tokyo 162–8601, Japan.*

*corresponding author: [yui@rs.tus.ac.jp](mailto:yui@rs.tus.ac.jp).Tel.: +81-3-5228-8728. Fax: +81-3-5228-9060.

**Fig. S1**  Frequency deviations for Raman bands of sulfur and silicon after the linear calibration. The error bars correspond to standard deviation.

**Fig. S2** Biaxial plot for (a,b) dolomites and (c,d) breunnerites collected from different mines. (a) and (c) are those of T and L modes whereas (b) and (d) are of ν_1_ and L modes.

**Table. S1** Relationship between Raman shift and Mg / (Mg + Fe) ratio which was previously reported for dolomite-ankerite solid solutions.

| T / cm^-1^ | L / cm^-1^ | Mg / (Mg + Fe) | reference |
| --- | --- | --- | --- |
| 167 | 285 | 0.46 | a |
| 168 | 289 | 0.57 | a |
| 169 | 287 | 0.42 | a |
| 171 | 291 | 0.66 | a |
| 175 | 300 | 0.92 | a |
| 176 | 298 | 0.90 | a |
| 176 | 298 | 0.91 | a |
|  |  |  |  |
| 170.08 | 288.79 | 0.54 | b |
| 176.07 | 298.66 | 0.83 | b |
| 175.05 | 297.86 | 0.83 | b |

^a^ Y. Kim, M. C. Caumon, O. Barres, A. Sall, and J. Cauzid, Spectrochim. Acta A Mol. Biomol. Spectrosc. 261, 119980 (2021).

^b^ N. Rividi, M. van Zuilen, P. Philippot, B. Menez, G. Godard, and E. Poidatz, Astrobiology 10, 293 (2010).


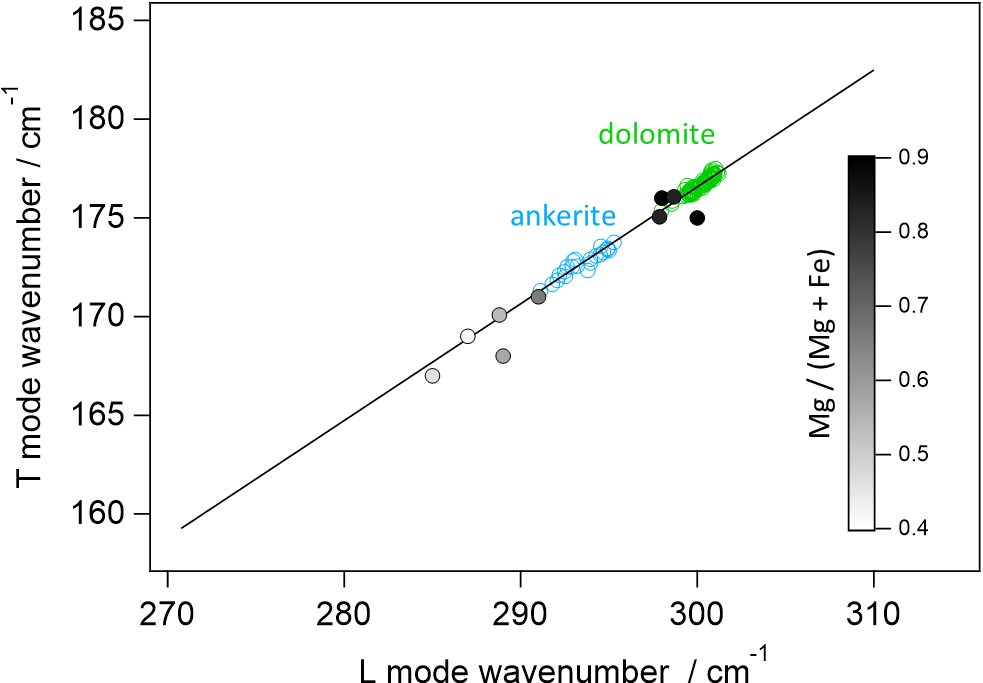


**Fig. S3** Biaxial plot for dolomite-ankerite solid solution series. The colored blank circles correspond to our data (same as those in Figs. 4b and 5 in the maintext), and black circles filled with grey correspond to the literature data shown in Table S1. For the literature data, those for Mg-rich samples are filled with darker-color. The color scale is shown as the inset.
